# Science, policy and trust: Lessons and future strategies for health crisis management in Norway

**DOI:** 10.1371/journal.pgph.0006787

**Published:** 2026-07-02

**Authors:** Guri Rørtveit, Karin Nygård, Bjørn Iversen, Christina Rolfheim-Bye, Preben Aavitsland

**Affiliations:** Norwegian Institute of Public Health, Oslo, Norway; Institute of Development Studies, UNITED KINGDOM OF GREAT BRITAIN AND NORTHERN IRELAND

## Background

The COVID-19 pandemic challenged governments across the globe. The course of the pandemic differed from country to country, leading to a range of governmental responses. Some implemented prolonged, strict lockdowns, while others adopted more flexible strategies that relied on individuals voluntarily taking measures to reduce the spread of the disease.

High levels of trust in public institutions and between levels of government facilitated cooperation and public compliance in Norway. Although Norway emerged from the pandemic with fewer health and economic losses than many other countries, there are still several lessons to be learned.

In Norway, the government, particularly the Ministry of Health and Care Services, played a leading role in setting national strategies and implementing measures to control the COVID-19 pandemic. The implementation of medical as well as public health and social measures in Norway has been described previously [1]. The decisions were to a large degree based on advice from expert agencies like the Norwegian Institute of Public Health (NIPH), the Norwegian Directorate of Health (DoH), and from the Regional Health Authorities. Early in the pandemic, most of the pandemic-related decisions were based on advice from the health sector. Later, the Ministry of Justice coordinated the broader governmental response through the Ministerial Crisis Council, ensuring cross-sectoral alignment and that other sectors such as education, police, transport, business, and culture were involved.

Norway’s decentralized governance also allowed municipalities significant autonomy. Municipal crisis teams including municipal Chief Medical Officers coordinated local responses, often adapting national guidelines to local contexts. NIPH provided municipalities with local surveillance data and a handbook with advice on control measures based on the local situation. DoH also gave advice, often more focused on legal issues. However, the roles between NIPH and DoH in providing advice were sometimes unclear.

Initially, there was some friction between national and local authorities, especially when local measures were stricter than those recommended nationally. Such tension also arose when national authorities decided or advised harmonization of measures across regions that had different infection rates. Over time, coordination between national and local authorities improved, with national strategies increasingly allowing for local adaptations. This balance between centralized guidance and local responsibility and flexibility in the municipalities was a key feature of Norway’s pandemic governance. NIPH implemented the COVID-19 vaccination program in close collaboration with the municipalities, further emphasizing the crucial role of primary health care in managing the pandemic in Norway.

Municipal response was less integrated in various plans at national level, resulting in, for example, a lack of personal protective equipment for primary health care in the beginning. Furthermore, the concept of “knowledge preparedness” was not a feature of preparedness plans before the pandemic. The need for rapid and reliable knowledge is crucial in times of uncertainty. Research studies and knowledge synthesis contributed heavily to the scientific evidence and expert advice which were central to Norway’s pandemic response.

NIPH played a pivotal role in producing knowledge, conducting surveillance and risk assessments, and advising the Ministry of Health, the municipalities and the health care services. Although NIPH’s role was advisory, its input significantly influenced policy decisions. NIPH was regularly represented in meetings at governmental, as well as ministerial level. In some cases, political decisions diverged from NIPH’s recommendations, reflecting the complex interplay between scientific advice and political considerations and priorities. The most prominent and debated example is the government’s decision on school closures [2,3] As the advice from NIPH and DoH was regularly published and available to the public, anyone could follow the risk analysis, modelling, surveillance data and advice provided to the government. Norway has, over many years, been characterised by a high level of public trust in governmental authorities. Although a pandemic may create conditions conducive to communication challenges and an erosion of trust, this was not observed. The transparency is considered to have contributed to a high level of trust throughout the pandemic, and may serve as a relevant example to other countries [4].

## Preparedness registry for knowledge production in crises

Surveillance and health registry data are invaluable sources of information for pandemic management. The 2009 H1N1 influenza pandemic showed the critical need to integrate and link health data with administrative and demographic information to assess intervention effectiveness and understand broader societal impacts. At the time, however, such integration lacked a clear legal framework, which led to long delays in producing research and knowledge relevant for crises management. In response, the Norwegian government revised the health preparedness legislation in 2016 to enable the establishment of emergency preparedness registers where data from different registers and sources can be linked by the personal identification number. This legal foundation was directly informed by the challenges encountered during the 2009 pandemic and aimed to strengthen crisis response and facilitate timely data and knowledge production during a crisis. The legislation clarified procedures for data collection and use during public health emergencies, ensuring compliance with privacy regulations while allowing for timely action. This legislation was a key element enabling the establishment of the preparedness registry during the COVID-19 pandemic and made real-time integrated data from several registries available for crisis management on a whole new scale.

In April 2020, the new legislation was used for the first time when NIPH in collaboration with DoH and the Norwegian Intensive Care and Pandemic Registry established the COVID-19 preparedness registry (Beredt C19). The purpose was to establish a registry that linked data from different sources, in order to monitor the development of the pandemic and generate knowledge to support evidence-based decision-making in managing the pandemic. Data sources included infectious disease surveillance data, microbiological data from laboratories, hospital and intensive care admissions, vaccination registry, primary care consultations, cause of death registry, occupation and social services data, contact tracing data and border entry registrations. The platform was developed with an emphasis on ensuring individual privacy and data protection by integrating safety mechanisms and procedures in the system.

The access to almost real-time data through the pandemic preparedness registry was crucial for managing the pandemic both on the local and the national level. Additionally, NIPH contributed to the global knowledge base. This was achieved not only using national data, but also by sharing, analysing, and publishing combined data from the Nordic countries. Important publications based on the preparedness registry include documentation proving that the COVID-19 vaccine was safe for pregnant women [5] and that vaccination increased the risk of myocarditis slightly among younger males [6].

The experience gained from establishing the Norwegian emergency preparedness registry is transferable to other countries. Although many countries already have well-developed registries, they may not necessarily have established the legal and technical frameworks required to implement such an emergency preparedness registry. We would strongly encourage the initiation of these processes wherever feasible – before the next health crisis occurs.

## Public trust as a basis for communication

In the early stages of a health crisis, uncertainty about the situation will be a dominant feature. Nevertheless, actions must be taken, and the population’s compliance is crucial for these actions to have an impact. To achieve this, trust is essential [7].

The most important factor in ensuring and enhancing trust in the authorities during a crisis is not necessarily the actions taken, but the behaviour that builds trust and trustworthiness in everyday life, between crises [8]. The authorities’ culture of transparency about processes and the knowledge base in everyday life fosters public expectations of the same level of transparency during a crisis. High trust at the onset of a crisis thus lays the foundation for openness about uncertainty and encourages open public debate. This characterized the pandemic communication in Norway.

As the pandemic, the virus, disease severity, knowledge and measures changed over time, NIPH and DoH worked closely together in developing and adapting the communication platforms that were most effective and appropriate at the time. WHO’s 2017 guide for public health crisis communication emphasizes trust-building and outlines eight key factors, including transparency, timely information, scientific clarity, public dialogue, and consistent messaging. This was at the core of the communication strategies together with the Norwegian Government’s communication platform principles [9]:

- Openness. In its communication with the citizens, the government shall be open, clear and accessible
- Participation. The government shall take advice from affected citizens and involve them in the formulation of policies and services
- Reaching all. The government shall see to it that relevant information reaches everyone concerned
- Active. The government shall actively and in due time inform about rights, obligations and opportunities
- Coherence. Government communication shall be perceived as comprehensive and coordinated
- Line management principle. Communication responsibility accompanies the case responsibility

In addition to weekly publications of reports on the ongoing status for the pandemic in Norway and continuous publications of risk assessments, NIPH engaged in debates with other agencies and the government about the pandemic, the possible strategies for managing it, and the measures put in place, sometimes with diverging views. While such openness can lead to confusion, it also invites the public into the space of uncertainty and scientific debate, fostering trust. However, we acknowledge that in some cultures, open discussions on scientific uncertainty may reduce trust instead of increasing it.

These insights about communication and trust during the pandemic may apply specifically to countries similar to Norway, where high, pre-existing trust in institutions and transparent processes played a crucial role. Governmental institutions in Norway, including NIPH, have invested in this trust over years through open internal and external discussions, especially when clear scientific knowledge is lacking, and by using multiple expert voices in media. Some of the public measures implemented during the pandemic, like the effect of school closures and lockdowns, lacked clear scientific evidence, and the uncertainties were discussed openly.

One example of transparency of an internal discussion, is to be read in this answer to the government about vaccine recommendations [10] in which we write “There is scientific disagreement within the Norwegian Institute of Public Health (NIPH) regarding the need for booster doses in this entire age group, as the evidence base is insufficient to assess the benefits and risks.” (Our translation to English). The statement was repeated in a press conference and generated several media stories.

## How NIPH is preparing for the next crisis

Knowledge preparedness is central to the NIPH strategy for readiness in the face of the next health crisis. Our experiences from the pandemic, some of which are described above, have made us identify five key components to improve robustness and resilience in knowledge preparedness, and our ability to quickly scaling up during a crisis. Although the details are national, the concepts are generalisable to other countries:

1. *Improved surveillance:* NIPH is working within a four-year perspective (2024–28) to improve routine surveillance of pathogens, infectious diseases, immunity, and vaccination, to strengthen Norway’s preparedness for future major epidemics or pandemics. Data will be collected more automatically and rapidly from the sources and compiled more efficiently, enabling surveillance results to be produced in a timely manner for those who need information to support situational awareness, decision-making, and prioritization. This is in line with the revised EU legislation that underpins the importance of national and international surveillance for crisis management.
2. *Flexible and scalable knowledge production and sharing*: NIPH is a major research institution with strong methodological expertise for producing reliable knowledge during a crisis. Building on our experience with the preparedness registry, we are establishing new infrastructure for knowledge production based on real-time registry data.
  Primary health care plays a key role as the first responder and manager of less severe cases. It is therefore essential to provide this part of the health system with timely data and relevant information. We have recently launched a dashboard with real-time data on communicable diseases and vaccine uptake for Chief Medical Officers in the municipalities, and we plan to expand this further (Kommunens sykdomsoversikt – KoSy - FHI, only available in Norwegian).
3. *Planning and rehearsals:* This involves collaboration across NIPH and with other institutions to be prepared when a crisis hits. In the revised national preparedness plans, knowledge preparedness has been included and highlighted as important in preparedness planning, for both the producers and the users of knowledge. Developing infrastructure to enable real-time access to data in a crisis is an essential part of this work. Another aspect of planning is having pre-approved protocols for research that can be conducted during a crisis.
4. *Establishment of networks:* This includes agreements with national academic institutions about preparedness and collaboration on knowledge production in crises, as well as securing and expanding existing national and international networks. Academic and international partnerships are already embedded in our daily activities, and the scientific cooperation between the Nordic countries that proved essential during the COVID-19 pandemic will be further strengthened. Networks should be established and actively collaborating between emergencies and have plans for rapidly scaling up in emergencies. The EU has launched several initiatives where NIPH is active, like the Pandemic Preparedness Partnership Be Ready Now and the European Vaccine Hub (EVH). NIPH also has a collaboration agreement with Coalition for Epidemic Preparedness Innovations (CEPI).
5. *Scientific independence:* To ensure trust in scientific advice, it is important for NIPH as a public health institute to have a legal framework, clearly describing roles, tasks and responsibilities. The institute’s scientific independence from political and other external interference in developing, producing, and disseminating scientific knowledge is an important element [11].

## New challenges for future health crises

During international epidemics and pandemics, the sharing of surveillance results and research findings is crucial for effective management of the situation. However, open sharing may be hindered if the geopolitical context is characterized by mistrust, hostility, or even open conflict between countries. Commercial interests may also obstruct information exchange.

Under the International Health Regulations and the new Pandemic Agreement, the World Health Organization (WHO) has both the mandate and a strong position to coordinate international information sharing. Some countries, including the United States, have currently suspended its collaboration with WHO, which may reduce the availability and exchange of critical information on emerging health threats [9]. We also observe tendencies toward weakened financing of WHO as well as of national centres for disease prevention and control, further decreasing the collective preparedness for a new pandemic.

Effective communication with the public is an essential tool for managing future pandemics and other health crises. At the core of effective communication lies transparency, the ability to listen to the public – and the understanding that trust goes two ways; the public’s trust in the authorities is affected by the authorities’ trust in the public. The ability to reach all groups in our society with well-founded and accurate information may be further challenged in the years to come. The channels and actors of disinformation seem to increase. Signs of politicization of science are visible in several countries, where political leaders shed doubt on scientific findings and facts. A shared effort within the health authorities to build trust using science and appropriate communication tools to counter disinformation will be more important than before and need continuous focus in the national and international public health institutions.

The pandemic provided extensive experience and important lessons. In this paper, we reflect on the lessons learned and identify several new challenges. Given the cross-border nature of pandemics and outbreaks, we believe these lessons and challenges are relevant to most countries and public health agencies. As each country strengthens its surveillance and knowledge generation, shares experiences, and learns from others while building on its own national strengths and addressing its specific challenges, the global community becomes more resilient.
